# Enhanced Antitumor Effect of Trastuzumab and Duligotuzumab or Ipatasertib Combination in HER-2 Positive Gastric Cancer Cells

**DOI:** 10.3390/cancers13102339

**Published:** 2021-05-12

**Authors:** Maria Maddalena Laterza, Vincenza Ciaramella, Bianca Arianna Facchini, Elisena Franzese, Carmela Liguori, Stefano De Falco, Paola Coppola, Luca Pompella, Giuseppe Tirino, Massimiliano Berretta, Liliana Montella, Gaetano Facchini, Fortunato Ciardiello, Ferdinando de Vita

**Affiliations:** 1 UOC Oncologia, ASL Napoli 2 Nord, P.O. “S.M. delle Grazie”, Pozzuoli-Ischia, 80078 Napoli, Italy; elisena.franzese@aslnapoli2nord.it (E.F.); carmenliguori.199@gmail.com (C.L.); stefano.defalco@aslnapoli2nord.it (S.D.F.); paola.coppola@aslnapoli2nord.it (P.C.); liliana.montella@aslnapoli2nord.it (L.M.); gaetano.facchini@aslnapoli2nord.it (G.F.); 2 Department of Precision Medicine, University of Campania ‘Luigi Vanvitelli’, 81100 Napoli, Italy; vincenza.ciaramella@unicampania.it (V.C.); biancaarianna.facchini@studenti.unicampania.it (B.A.F.); luca.pompella@unicampania.it (L.P.); giuseppe.tirino@unicampania.it (G.T.); fortunato.ciardiello@unicampania.it (F.C.); ferdinando.devita@unicampania.it (F.d.V.); 3 Department of Clinical and Experimental Medicine, University of Messina, 98121 Messina, Italy; mberretta@unime.it

**Keywords:** PI3K, AKT, HER3, ipatasertib, duligotuzumab, trastuzumab, gastric cancer

## Abstract

**Simple Summary:**

The ToGA trial has demonstrated, in HER2-expressing patients, that unresectable and advanced gastric cancer, chemotherapy and trastuzumab in combination increase overall survival, even if it is still unclear why after one year the same patients are non-responsive to trastuzumab treatment. Here, we have demonstrated that in HER2-positive gastric cancer cell lines, the addition of duligotuzumab, targeting HER3 receptor, or ipatasertib, targeting AKT protein, enhances the antitumor effect of trastuzumab in vitro through a full inhibition of the membrane signals, on HER2 and HER3, and of downstream signaling, including AKT, and MAPK pathways. Hence, this study suggests a novel and biomarker-driven therapeutic strategy supporting further evaluation of the anti-tumor efficacy of these combinations in HER2 human gastric cancer patients.

**Abstract:**

The anti-HER2 monoclonal antibody trastuzumab is a key drug for the treatment of HER2-positive gastric cancer (GC); however, its activity is often limited by the onset of resistance and mechanisms of resistance are still poorly understood. Several targeted agents showed synergistic activity by concomitant use with trastuzumab in vitro and are under clinical investigation. The aim of this study was to assess the antitumor activity of duligotuzumab, an anti HER3/EGFR antibody or ipatasertib, an AKT inhibitor, combined with trastuzumab in a panel of HER2-positive human gastric cancer cells (GCC), and the efficacy of such combinations in HER2-resistant cells. We have assessed the efficacy of duligotuzumab or ipatasertib and trastuzumab in combination, analyzing proliferation, migration and apoptosis and downstream intracellular signaling in vitro on human HER2-positive GCC (NCI-N87, OE33, OE19) and in negative HER2 GCC (MKN28). We observed a reduction of proliferation, migration and apoptotic rate in HER2-positive OE33, OE19 and N87 cell lines with the combination of duligotuzumab or ipatasertib plus trastuzumab. In particular, in OE33 and OE19 cell lines, the same combined treatment inhibited the activation of proteins downstream of HER2, HER3, AKT and MAPK pathways. Targeting both HER2 and HER3, or HER2 and AKT, results in an improved antitumor effect on HER2-positive GCC.

## 1. Introduction

Gastric cancer is the fourth most common malignant neoplasm and the second leading cause of cancer-related death in Western countries [1]. Although the use of last generation chemotherapy regimens has improved survival in patients with gastric cancer, the prognosis of unresectable or metastatic gastric cancer patients remains poor, with a median overall survival of 12 months and a 5-year survival < 7% [2,3].

Significant progresses in the comprehension of pathogenesis of gastric cancer and a better knowledge of its biological heterogeneity have led to the identification of new targets and to the development of new molecular drugs [4].

Trastuzumab, a recombinant humanized monoclonal antibody that targets the extracellular domain IV of HER2, is the first and only molecular target drug approved for the treatment of advanced HER2-positive gastric cancer [5,6,7].

The ToGA trial has shown that trastuzumab plus conventional chemotherapy has demonstrated a significant clinical benefit compared to chemotherapy alone in terms of response rate, progression-free survival (PFS) and overall survival (OS), and has therefore become the new standard of treatment for metastatic HER2-positive gastric cancer [8].

However, some evidence indicates that the efficacy of trastuzumab plus chemotherapy may actually be less than expected on the basis of the results of the ToGA trial in actual clinical practice [9].

Further clinical trials conducted with other anti-HER2 agents such as lapatinib, TDM-1 and pertuzumab, failed to show a survival advantage, compared to standard first- or second-line treatment [10,11,12].

Several preclinical studies have investigated the mechanisms of resistance to trastuzumab, in order to identify new molecular targets in HER2-positive gastric cancer patients [13].

In particular, activation of the PI3K/AKT signaling pathway and enhanced signaling from HER family receptors, including overexpression of HER3, are involved in mediating trastuzumab resistance in HER2-positive GC cells [14,15,16,17,18,19].

Thus, selective PI3K- or AKT-inhibitors and anti-HER3 antibodies represent a novel option to overcome resistance to anti-HER2 therapy and improve gastric cancer prognosis. To date, a number of drug candidates targeting HER3 and PI3K/AKT pathways are under clinical evaluation for the treatment of gastric cancer [20,21,22].

Ipatasertib (GDC-0068) is an oral, investigational small molecule ATP-competitive with the potential to inhibit of all three isoforms of AKT [23].

In preclinical models of gastric cancer cells, ipatasertib acts synergistically with fluorouracil (5-FU) and platinum chemotherapy. A clinical trial aiming to evaluate the efficacy of ipatasertib in combination with FOLfox6 in (NCT01896531) HER2-negative metastatic gastric cancer patients is ongoing [24].

Duligotuzumab (MEHD, MEHD7945A) is a novel humanized IgG1 antibody that is directed at HER3 and EGFR, which is under investigation in phase I and II trials for metastatic colon rectal cancer and squamous cell carcinoma of the head and neck [25,26].

The aim of our study is to investigate the efficacy of duligotuzumab or ipatasertib in combination with trastuzumab by evaluating in vitro the antiproliferative, pro-apoptotic and anti-migration effects in HER2-positive gastric cancer cells.

## 2. Results

### 2.1. Effect of Duligotuzumab or Ipatasertib in Combination with Trastuzumab on Cell Proliferation in a Panel of Human Gastric Cancer Cell Lines

The antiproliferative effects of duligotuzumab or ipatasertib as single agents or in combination with trastuzumab was evaluated in a panel of Her2 positive or negative human gastric cancer cell lines with different biologic profiles.

MKN28 gastric cells are human gastric adenocarcinoma-derived cell lines and were selected as a HER2-negative model. Among HER2-positive human gastric cancer cell lines, we chose three cancer cell lines: NCI–N87, 0E19 and OE33. Cell proliferation was measured with the 3-(4,5- dimethylthiazol-2-yl)-2,5 diphenyltetrazolium bromide (MTT) assay. As anti-HER2 agent, we selected trastuzumab, currently used for the treatment of metastatic gastric HER2-positive cancer patients. Different doses of ipatasertib, duligotuzumab, trastuzumab alone and the combination of trastuzumab with duligotuzumab or ipatasertib were tested.

**Figure 1:**
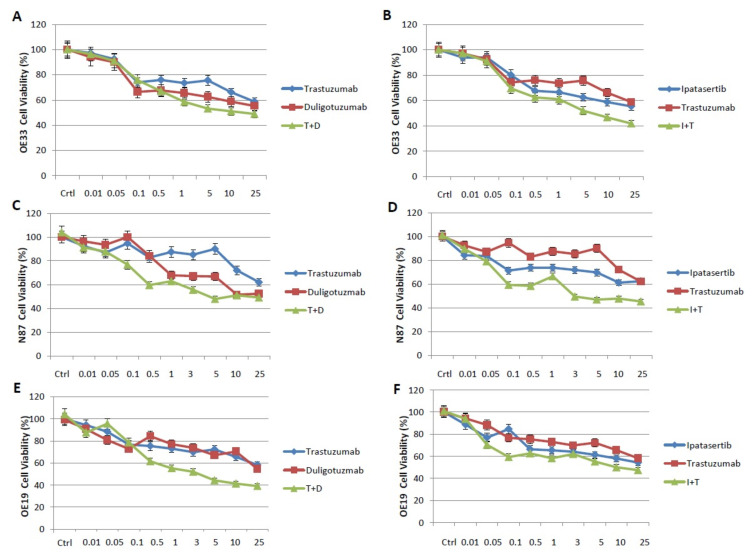
Effect of Trastuzumab, Ipatasertiband Duligotuzmabon cellproliferationin a panel of human gastriccancercelllines: OE33 (**A**,**B**), N87 (**C**,**D**) and OE19 (**E**,**F**). Cellsweretreatedwith differentconcentrationof eachdrug, assingle agent and in combination(Trastuzumaband Duligotuzumab, T+D; Ipatasertiband Trastuzumab, I+T), for 72 h and evaluatedfor proliferationby MTT staining, asdescribedin Materialsand Methods. The resultsare the medianof threeseparate experiments, eachperformedin triplicate.

**Figure 2:**
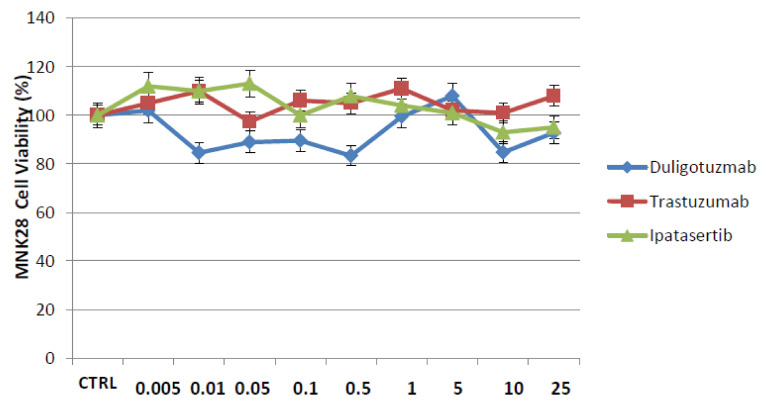
Effect of Trastuzumab, Ipatasertib and Duligotuzmab on cell proliferation in MNK28, human gastric cancer cell line, treated with different concentration of each drug for 72 h and evaluated for proliferation by MTT staining, as described in Materials and Methods. The results are the median of three separate experiments, each performed in triplicate.

From the dose response curve results, using doses from 0.01 to 25 µM for each drug, IC50 values ranged from ≈0.1 to ≈0.5 µM for duligotuzumab and ipatasertib, whereas the IC50 values for trastuzumab ranged from ≈0.5 to ≈1 µM (Figure 1, Table S1) The same experiment was also performed on the cell line MNK28 and the curves show that there is no effect on cell proliferation by demonstrating that there is no response in HER2 negative cells (Figure 2).

Combined treatment of duligotuzumab or ipatasertib and trastuzumab resulted in an important antiproliferative effect as compared to single agents all of HER2 gastric cancer cells (Figure 1A–F).

### 2.2. Effects of Duligotuzumab, Ipatasertib and Trastuzumab on Colony Formation and Migration Abilities of Human Gastric Cancer Cell Lines

**Figure 3:**
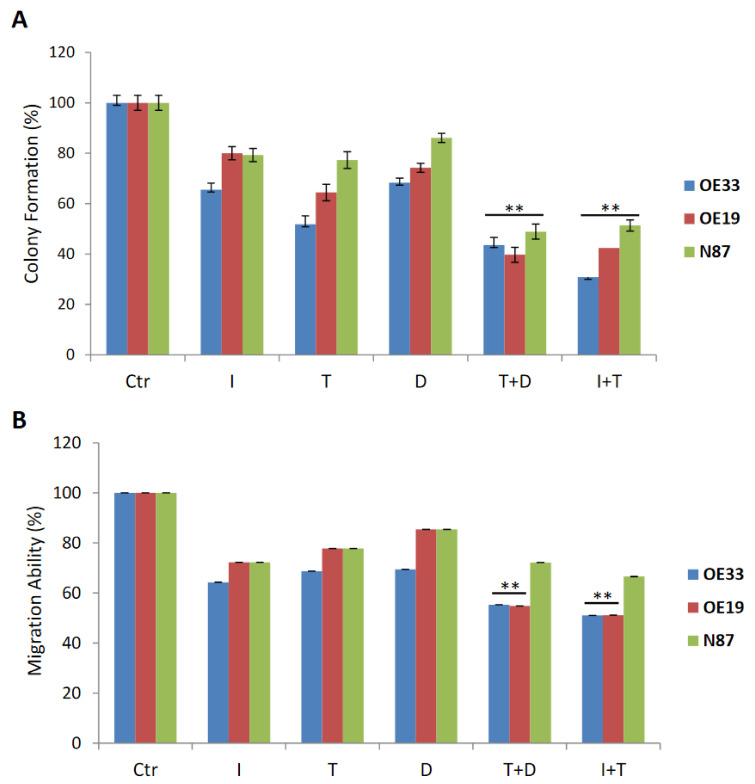
Effecton colonyformation (**A**) and migrationability (**B**) of Trastuzumab, Ipatasertiband Duligotuzmabtreatment, assingle agent and in combination, in human gastriccancercelllines: OE33, OE19 and N87. (C: untreatedcontrol, T: Trastuzumab, D: Duligotuzumab, I: Ipatasertib, Trastuzumabwith Duligotuzumab, T+D; Ipatasertibwith Trastuzumab, I+T). Eachassaywasperformedasdescribedin Materialsand Methods, afterthe indicatedtreatmentsatIC50 doses. The resultsare the average±SD of threeindependentexperiments, eachdonein triplicate. Asterisksindicate statisticalsignificance (** *p* ≤ 0.01).

The synergistic effect of the two drugs is evident with dose of 0.5 µM and therefore this dose was chosen for the subsequent experiments. The experiments were conducted on the two cell lines that showed higher response: OE19 and OE33 cells. For this reason, cells were treated with duligotuzumab, ipatasertib alone or in combination with trastuzumab observing that cells that manage to retain the ability to migrate to and form colonies with the single agent reach 70–80%, while the combination of two drugs, duligotuzumab plus trastuzumab and trastuzumab plus ipatasertib, drastically reduces these cell abilities under 40% of the initial population (Figure 3).

### 2.3. Apoptosis Analysis of Human Gastric Cell Lines after Treatment with Duligotuzumab, Ipatasertib and Trastuzumab

**Figure 4:**
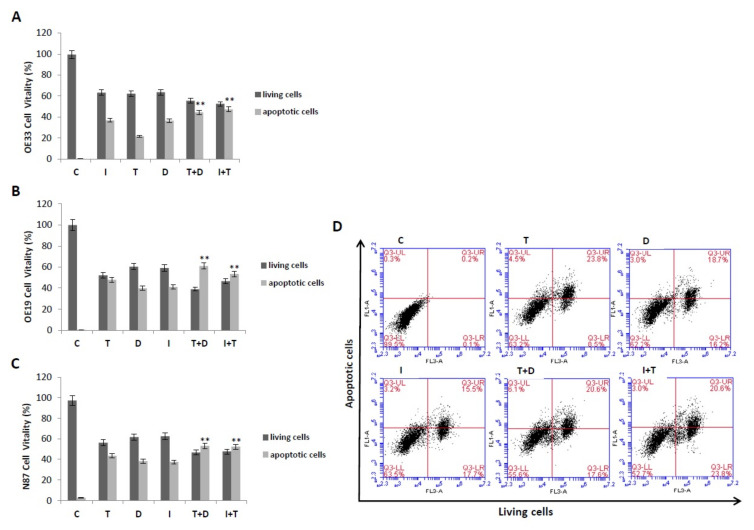
Flow cytometric analysis of OE33 (**A**), OE19 (**B**) and N87 (**C**) cell apoptosis after treatment with Trastuzumab, Ipatasertib and Duligotuzmab, as single agent and in combination. One representative experiment is shown. Dot plot diagrams shown the different stages of apoptosis: % indicated in the upper quadrant represent cells positive for Annexin V, % in lower quadrant represent viable cells. In the histogram plot, dark column corresponds to living cells and clear column to apoptotic cells. (C: untreated control, T: Trastuzumab, D: Duligotuzumab, I: Ipatasertib, Trastuzumab with Duligotuzumab, T+D; Ipatasertib with Trastuzumab, I+T). Each of them represents mean values obtained from three separate experiments. Asterisks indicate statistical significance (** *p* ≤ 0.01).

Apoptosis analysis was performed after 72 h treatment with duligotuzumab or ipatasertib and trastuzumab combination in NCI–N87, 0E19, OE33 human gastric cancer cell lines. As shown in Figure 4, flow cytometric analysis obtained that treatment with duligotuzumab or ipatasertib and trastuzumab in combination significantly increased by several folds the percentage of apoptotic cells in all the cell lines tested. In particular, OE33 cells presented respectively ≈35% apoptotic rate in duligotuzumab, ipatasertib and trastuzumab (at single doses of 0.5 µM respectively), while the combination treatments reached ≈60% of apoptotic cells with trastuzumab plus duligotuzumab or ipatasertib, respectively (Figure 4A). Similar effects have been showed in the other two cell models (Figure 4B,C).

### 2.4. Protein Analysis of Intracellular Signaling Pathways in Human Gastric Cancer Cell Lines

**Figure 5:**
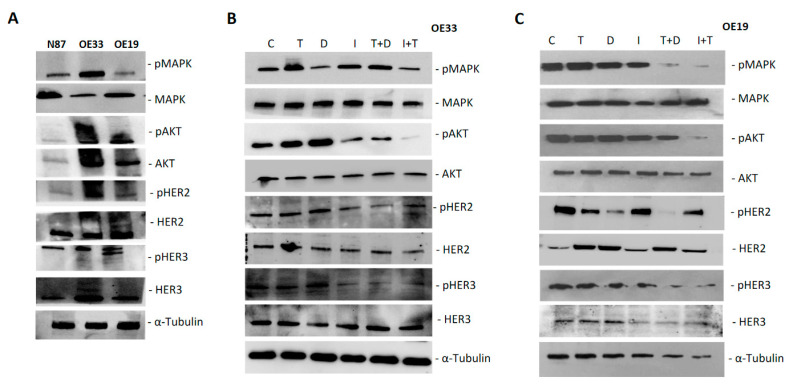
Protein analysis on lysates from N87, OE33 and OE19 cell lines with indicated antibodies (**A**). Western blotting analysis of intracellular proteins and their phosphorylated isoforms following treatment with Trastuzumab, Ipatasertib and Duligotuzmab, as single agent and in combination in OE33 (**B**) and OE19 (**C**). (C: untreated control, T: Trastuzumab, D: Duligotuzumab, I: Ipatasertib, Trastuzumab with Duligotuzumab, T+D; Ipatasertib with Trastuzumab, I+T). α Tubulin was included as a loading control.

Western blot analyses were performed to evaluate the effect on intracellular signaling pathways on protein extracts from OE33 and OE19 cell lines after 48 h treatment with duligotuzumab or ipatasertib and trastuzumab, at IC50 doses from cell growth inhibition tests, as single agents or in combination (Figure 5).

First, the expression of specific proteins in extracts of each cell line was verified, in particular the presence of all the markers involved in the signaling mechanism of PI3K/AKT and of HER2 and HER3 receptors, in both total and phosphorylated isoform. Then, Western blot analysis was performed following treatments to investigate the variation of these markers with single agents and combinations (Figure 5A).

**Figure 6:**
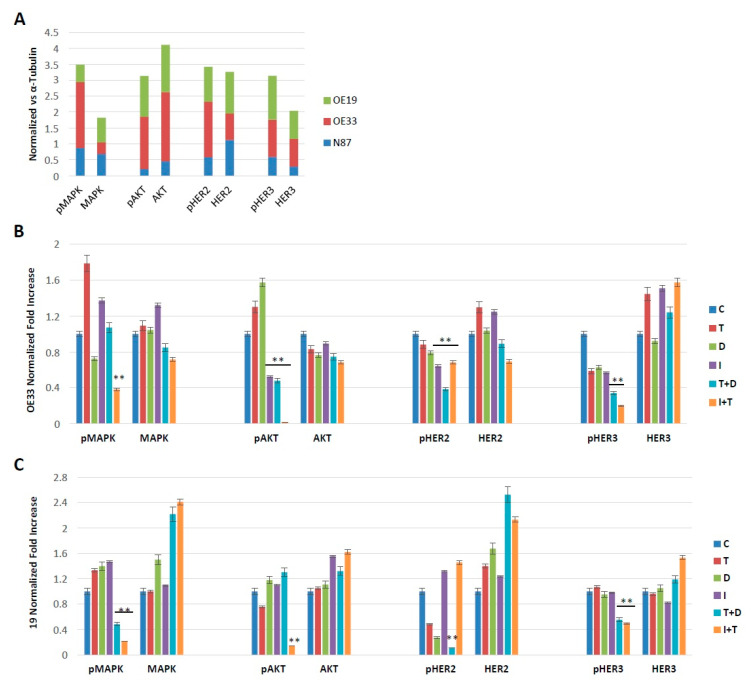
Graphic representation of western blotting data (shown in Figure 5) by densitometric analysis and relative normalization of each band compared with α Tubulin (C: untreated control, T: Trastuzumab, D: Duligotuzumab, I: Ipatasertib, Trastuzumab with Duligotuzumab, T+D; Ipatasertib with Trastuzumab, I+T). Asterisks indicate statistical significance (** *p* ≤ 0.01).

We found that single agents, duligotuzumab, ipatasertib and trastuzumab, have no effect on the expression of AKT and MAPK proteins, while the combination of two drugs, duligotuzumab plus trastuzumab and trastuzumab plus ipatasertib, resulted in a strong reduction in phosphor-AKT and phosphor-MAPK, leaving the expression of the total forms of AKT and MAPK unchanged both in KATOIII and in OE19 cell lines, thus demonstrating a downstream effect on the PI3K/AKT pathway. A similar “switch-off” effect was also observed in the expression of HER2 and HER3 receptors, which were reduced by the combination of the two drugs in both cell lines, though most significantly in the OE19 cell line (Figure 5B,C). The results of the images were quantified by comparison with tubulin, a constitutively expressed protein and analyzed by Image J software to evaluate the significance of the differences (Figure 6).

## 3. Discussion

The phase III ToGA trial proved the superiority of trastuzumab, plus standard chemotherapy in patients with HER2-positive metastatic gastric cancer compared to chemotherapy alone in terms of OS, PFS and response rates. However, the objective response rate to trastuzumab combined with chemotherapy failed to reach 50% in a real-practice setting, with a median PFS of 6.7 months [9].

The subsequent randomized phase III trials evaluating other anti-HER2 agents such as lapatinib or TDM-1 in this setting of patients failed to show any clinical benefit compared to chemotherapy alone [10,11,12].

Finally, the addition of pertuzumab to chemotherapy plus trastuzumab was also not associated with a survival advantage compared to trastuzumab plus chemotherapy [27].

Based on these data, there is a compelling need to explore resistance mechanisms associated with anti-HER2 therapy and to establish rational strategies to improve trastuzumab efficacy in HER2-positive GC patients [19,20,21,22,23,24,25,26,27,28].

In a study designed to explore potential causes of failure of anti-HER2 agents in HER2-positive GE adenocarcinomas, Kim et al. observed that the majority of HER2-amplified tumors harbored secondary oncogenic alterations involving genes related to cell-cycle regulation such as PI3K pathway mutations and receptor tyrosine kinase (EGFR, HER3, MET) amplifications. Several studies have demonstrated that somatic mutations in PI3KCA/AKT and in HER3 are present respectively in 4–25% and 16–59% of patients with gastric carcinoma [29,30].

This evidence suggests that additional oncogenic alterations may represent the key molecular features responsible for the lack of benefit derived from HER2 inhibition in patients with HER2-positive GC [31].

In the present work, we developed a preclinical model using human HER2-positive gastric cancer cells in order to assess the activity of trastuzumab in combination with the AKT-inhibitor ipatasertib or the anti-HER3 antibody duligotuzumab. We reported evidence supporting the improved anti-proliferative in vitro effect of the combination vs. single agent treatment in multiple human gastric cancer cell lines. In particular, we demonstrated that the combination of duligotuzumab or ipatasertib, plus trastuzumab, induced a stronger inhibition of cell proliferation compared to single treatment alone by using the 3-(4,5- dimethylthiazol-2-yl)-2,5 diphenyltetrazolium bromide (MTT) assay in HER-2 positive, but not in HER2-negative cell lines.

The synergistic effect of trastuzumab combined with different anti-HER3 antibodies, such as 1A 5 -3D4, and AKT inhibitors such as MK-2206, has been confirmed in several HER2-positive GC cell lines [32,33,34].

Our work showed that combination treatments resulted in reduced migration and invasion of gastric cancer cells in vitro, with an increased percentage of apoptotic cells in all cell lines tested by flow cytometric analysis. For instance, in OE33 and OE19 cells, the apoptotic rate increased from 35% to 60% when duligotuzumab or ipatasertib were combined with trastuzumab.

Additionally, immunoblotting analysis showed that while the expression of the total forms of AKT and MAPK were unchanged in OE33 and OE19 cell lines, a strong reduction in phosphor-AKT and phosphor-MAPK levels was observed only with the combination of duligotuzumab or ipatasertib plus trastuzumab, thus demonstrating an improved inhibitory effect on the PI3K/AKT pathway. A similar “switch-off” effect was also observed by assessing expression levels of HER2 and HER3 receptors. A more marked effect associated with combination treatment was obtained in the OE19 cell line, probably because of the higher HER2 expression level of these cells, which translates in a stronger HER2 activity of this model. Taken together, these results suggest that a complete inhibition of HER3, AKT and MAPK signaling pathways is needed for an optimal antitumor effect of trastuzumab.

This study suffers from multiple limitations, including the limited number of HER2-amplified gastric cancer cell lines, which makes our finding difficult for the generalization of our findings. Furthermore, our study did not evaluate simultaneously the effect of these combinations on trastuzumab-resistant HER2-positive gastric cancer cell lines, although experiments in this setting are ongoing.

Finally, our findings demonstrate that inhibition of other pathways may enhance trastuzumab sensitivity in HER2-amplified gastric cancer cells, suggesting the potential for combinational strategies in this setting.

## 4. Materials and Methods

### 4.1. Cell Lines and Drugs

The human gastric cancer OE33, NCI-N87 and MNK28 cell lines were provided by American Type Culture Collection (ATCC, Manassas, VA, USA) and OE19 by Sigma Aldrich (Saint Louis, MO, USA). Cells were maintained in stable humid conditions at 37 °C with 5% CO_2_ and cultured with Dulbecco’s modified Eagle’s medium (DMEM) supplemented with 20% fetal bovine serum (FBS; Life Technologies, Gaithersburg, MD, USA) and 1% antibiotics/antimycotics (Life Technologies, Gaithersburg, MD, USA) and Roswell Park Memorial Institute (RPMI, Sigma-Aldrich, Saint Louis, MO, USA) medium added with 10% FBS and 1% antibiotics/antimycotics, OE33/OE19 and NCI-N87/MNK28, respectively.

STR profiling (Promega) was used to confirm the identity of all cell lines prior to performing experiments, and was repeated after the majority of the experiments were performed.

Each cell line contains genomic mutations in one or more of the following genes according to the Sanger COSMIC database: CDKN2A, TP53, SMAD4, CDH1, CTNNB1, KRAS, MLH1 and PIK3CA/AKT (8–11%). The OE19 cell line contains a 100-fold amplification of the ERBB2 gene, and highly expresses its messenger ribonucleic acid. OE33, has an ERBB2 amplification of a lesser copy number. The NCI -87 cell line expresses levels of c-myc and c-erb-B 2 RNA that is comparable to other cell lines. Trastuzumab, duligotuzumab and ipatasertib, provided by Roche Genentech, were dissolved in sterile dimethylsulfoxide (DMSO) while working concentrations were prepared in culture medium just before each experiment.

### 4.2. Cell Proliferation Assays

Cancer cells were seeded in 96-multiwell plates (10,000 cells per well) and treated for 72 h with an increasing range of concentrations of each drug to determine the IC_50_.

The proliferation rate was measured with the MTT assay, as previously described. The results showed the median of three separate experiments, each performed in quadruplicate. Synergism was calculated with ComboSyn software, ComboSyn Inc., Paramus, NK 07652, USA.

### 4.3. Colony-Forming Assays

The assay was performed in 6-well tissue culture dishes where cells were distributed at 300 cells per well and treated with duligotuzumab, trastuzumab and ipatasertib. All conditions were performed in triplicate using untreated cells as reference control. Cells were cultivated for 7 days, at which point they were fixed with 4% paraphormaldeid, stained with crystal violet and colonies counted using the GelCount (Oxford Optronix, Abingdon, UK) [35].

### 4.4. Migration Assays

The in vitro invasive ability of cancer cells was analyzed by using 24-well Transwell chambers (Corning Life Sciences, Tewksbury, MA, USA) according to the manufacturer’s protocol [36]. Briefly, cells were seeded onto the membrane of the upper chamber of the Transwell chambers and were treated with the indicated concentrations of drugs for 72 h. The medium in the upper chamber was serum-free, the medium at the lower chamber was filled with a normal growth medium containing 10% FBS as a source of chemo-attractants. Cells that passed through the Matrigel-coated membrane were stained with Cell Stain Solution containing crystal violet (Chemicon, Millipore, CA, USA) and absorbance was measured by spectrophotometer after dissolving of stained cells. Untreated cells served as control and assays were performed in triplicate.

### 4.5. Evaluation of Apoptosis

Apoptosis assay was performed by flow cytometry according to the manufacturer’s instruction (Alexa Fluor 488 Annexin V/Dead Cell Apoptosis Kit, Invitrogen Carlsbad, CA, USA). Early apoptotic cells (Annexin V–positive, propidium iodide–negative), necrotic/late apoptotic cells (double positive), as well as living cells (double negative) were detected by FACS Calibur flow cytometer and then analyzed by Cell Quest software (Becton Dickinson Franklin Lakes, NJ, USA). Argon laser excitation wavelength was 488 nm, whereas emission data were acquired at wavelength 530 nm (FL-1 channel) for FITC and 670 nm (FL-3 channel) for propidium iodide.

### 4.6. Protein Expression Analysis

Following treatment, cancer cells were lysed with RIPA buffer (0.1% sodium dodecylsulfate (SDS), 0.5% deoxycholate, 1% Nonidet, 100 mmol/L NaCl, 10 mmol/L Tris–HCl (pH 7.4), 0.5 mmol/L dithiotritol, and 0.5% phenylmethyl sulfonyl fluoride, protease inhibitor cocktail (HoffmannLa Roche, Basilea, Svizzera) and isolated by centrifugation at 14,000 rpm for 10 min at 4 °C. After Bradford assay quantification (Bio-Rad Hercules, CA, USA), protein lysates were subjected to SDS-PAGE and Western blot analysis.

Primary antibodies for western blot analysis against p-EGFR (Tyr1068), EGFR, p-MAPK44/42 (Thr202/Tyr204), MAPK44/42, p-AKT (Ser473), AKT, pHER2 (Tyr1221/1222), HER2, pHER3 (Tyr1289) and HER3 were obtained from Cell Signaling Technology; monoclonal anti-α-tubulin antibody (T8203) from Sigma Chemical Co. St. Louis, MO, USA. The following secondary antibodies from Bio-Rad were used: goat anti-rabbit IgG and rabbit anti-mouse IgG. Immunocomplexes were detected with the enhanced chemiluminescence kit ECL plus (Thermo Fisher Scientific, Rockford, IL, USA) using the ChemiDoc (Bio-Rad). Each experiment was done in triplicate.

### 4.7. Statistical Analysis

Results are expressed as means ± s.d. from three independent experiments. Differences between groups were assessed by one-way analysis of variance (ANOVA). For all analyses *P* values represent 2-sided tests of statistical significance effects.

## 5. Conclusions

To date, trastuzumab remains the only biologic target agent approved for the treatment of HER2-positive metastatic gastric cancer. In the current study we have demonstrated that in HER2-positive gastric cancer cell lines the addition of duligotuzumab, targeting HER3 receptor, or ipatasertib, targeting AKT protein, enhances the antitumor effect of trastuzumab in vitro through a fully inhibition of the membrane signals, on HER2 and HER3, and of downstream signaling, including AKT, and MAPK pathways. These results suggest that targeting both HER2 and HER3 or HER2 and AKT provide a biological rationale for further evaluation of the anti-tumor efficacy of these combinations in HER2 human gastric cancer patients.

## Supplementary Materials

The following are available online at https://www.mdpi.com/article/10.3390/cancers13102339/s1, Table S1: IC doses for cell growth inhibition of single treatment with Trastuzumab, Ipatasertib and Duligotuzumab.
