# Supplementary material for: Enhanced Antitumor Effect of Trastuzumab and Duligotuzumab or Ipatasertib Combination in HER-2 Positive Gastric Cancer Cells
Source: Cancers (Basel). 2021 May 12;13(10):2339. doi: 10.3390/cancers13102339 (PMC8150287; doi:10.3390/cancers13102339)
Supplement: Supplementary file 1 [file cancers-13-02339-s001.zip › cancers-1182728-supplementary.pdf]

Table S1. IC doses for cell growth inhibition of single treatment with Trastuzumab, Ipatasertib and Duligotuzumab.

Cell Line	IC_{50} Trastuzumab	IC_{50} Ipatasertib	IC_{50} Duligotuzumab
**OE19**	≈ 25 µM	≥ 25 µM	≥ 25 µM
**OE33**	≈ 25 µM	5 µM	≈ 25 µM
**N87**	≥ 25 µM	≥ 25 µM	25 µM
